# Consequences, measurement, and evaluation of the costs associated with adverse drug reactions among hospitalized patients in China

**DOI:** 10.1186/1472-6963-14-73

**Published:** 2014-02-17

**Authors:** Shi Qing-ping, Jiang Xiao-dong, Ding Feng, Liu Yan, Yu Mei-ling, Zhu Jin-xiu, Zhang Shu-qiang

**Affiliations:** 1 Department of Pharmacy, the first Affiliated Hospital of Bengbu Medical College, 287 Zhihuai Road, Bengbu 233004, China; 2 Education and Research Center, Bengbu Medical College, Bengbu 233000, China; 3 Center of Adverse Drug Reaction monitoring, Bengbu Food & Drug Administration, Bengbu 233000, China

**Keywords:** Adverse drug reaction, Direct medical cost, Hospitalized patients, Tertiary care teaching hospital, Pharmacoeconomic

## Abstract

**Background:**

Adverse drug reactions (ADRs) are a leading cause of morbidity in developed countries and represent a substantial burden on health-care resources. Many countries spent 15% to 20% of their hospital budgets to treat drug complications. However, few studies have measured the pharmacoeconomic effects of ADRs on hospitalized patients in China. The study estimates the costs of ADRs as identified from the spontaneous voluntary reports completed from healthcare professionals. To do so, we calculate these costs, determine the sum of Medicare payments and their proportion of total healthcare spending, and evaluate the incidence of ADRs, characteristics of hospitalized ADR patients, and outcomes of ADRs in China.

**Methods:**

This retrospective survey studied patients who experienced ADRs during their hospitalization at a Chinese tertiary-care teaching hospital. The patients were divided into group A and group B according to general ADRs and serious ADRs in *Provisions for Adverse Drug Reaction Monitoring and Reporting*. The direct costs included treatment fees, inspection fees, laboratory fees, materials fees, bed charges, drug charges, nursing care, meals, and other expenses and the sunk-cost losses were calculated according to the hospital information system (HIS). Indirect costs of ADR treatment were calculated according to the human capital approach. The epidemiological characteristics of ADRs were evaluated.

**Results:**

2739 were diagnosed with ADR during the study period, which translates to an ADR rate of 0.81%. The total socioeconomic loss from 2739 cases of ADR was estimated at ¥817401.69, consisting of direct costs of ¥603252.81 and indirect costs of ¥214148.88. On average, the costs per patient amounted to ¥196.10 in group A, ¥7032.29 in group B. The sum of medicare payment and proportion were ¥219061.13 (65.23%) and ¥105422.02 (39.42%) in group A and B. The ADR incidence in old-age patients was significantly higher than in other age groups (*P* < 0.0001). The most common drug class associated with ADRs represented antibiotics (957 patients, 34.94%).

**Conclusions:**

The costs of especially severe ADRs could not be ignored, and in this hospital 0.13% of patients were diagnosed with ADRs associated with relatively higher direct costs than who suffered from mild ADRs, largely due to extended hospitalization.

## Background

It is estimated that adverse drug reactions (ADRs) ranked fourth to sixth on the factors that caused death in the United States; every year, hundreds of thousands of deaths directly resulted from ADR [1]. In many countries, ADRs accounted for more than 10% of the total number of hospitalization cases [2]. The treatment of diseases caused by ADRs requires a huge amount of financial resources, and many countries spend 15% to 20% of hospital budgets to treat drug complications [3,4]. Johnson and Bootman [5] show that the United States spends about $30.1 to $136.8 billion each year to deal with drug-related problems (DRPs). All the uncertainty associated with their estimations was evaluated by many scholars. This estimate was updated by Ernst and Grizzle to $177.4 billion for the year 2000 [6]. Of this amount, health-care-related drug costs accounted for the biggest part. Monitoring and analyzing ADR treatment costs has become a key research topic in Europe and the United States in recent years [7,8]. Hoonhout [9] and colleagues estimate that direct medical costs in Dutch hospitals amounted to euro 355 million per annum for all AEs and euro 161 million for preventable AEs in 2004.

China witnessed the beginnings of an ADR reporting and monitoring system in 1999. In 2011, this was upgraded to a comprehensive spontaneous reporting system (SRS) [10]. All units and individuals engaged in drug production, management, and usage in the country must report all suspicious ADRs and adverse drug events (ADEs) through the SRS. Since October 2003, hospitals in China have had a spontaneous ADR reporting unit, coordinated by their pharmacy practice departments. ADR reporting units of hospitals are peripheral centers under the National Pharmacovigilance Program. Through the SRS and the National Pharmacovigilance Program, the State Food and Drug Administration regularly publishes information on adverse drug reaction and revocation of drug approvals [11,12]. We can now provide a data infrastructure for ADR research with the accumulated ADR statistics. However, few pharmacoeconomics research studies on ADRs have examined the incidence of ADRs in China or the epidemiological characteristics of the disease. The objectives of this study are to (1) calculate the costs associated with different degrees of ADRs and determine total Medicare payments on ADRs as well as their proportion of total healthcare spending and (2) evaluate the incidence of ADRs, characteristics of hospitalized ADR patients, and outcomes of ADRs.

## Methods

### Setting

In this retrospective, descriptive research based on data derived from a spontaneous reporting system, we collected data of all cases of ADR that occurred during inpatient procedures at the First Affiliated Hospital of Bengbu Medical College from January 1, 2008 to December 31, 2012. All ADR cases reported to the database of the Center of Adverse Drug Reaction Monitoring through the SRS were retrospectively analyzed. Cases of ADR induced by drug-drug interactions were also included in the study. The following data were obtained from the clinical records of patients who suffered from ADR: (1) demographic information of patients, such as sex and age, medications taken prior to admission, history of drug allergy; (2) the sequence of ADR events and incidental information, such as time of occurrence, time of adoption of intervention measures to ADR , termination time of the ADR, the symptoms, signs, and related diagnostic findings when the ADR first occurred, therapy duration, and drug classes involved; (3) variation in symptoms, signs, and related diagnostic findings, the results of intervention measures, etc. The hospital is a large teaching tertiary hospital with 1936 beds. All the ADRs were re-evaluated according to the *Provisions for Adverse Drug Reaction Monitoring and Reporting*[13] issued in 2011, as well as the Naranjo scoring algorithm [14]. The drugs involved in the ADRs were codified into various drug classes according to their anatomical therapeutic chemical (ATC) classifications based on the World Health Organization (WHO) ATC Index of 2005 [15]. To address the issue of redundant and variable drug nomenclature, ADR names were coded by using the preferred terms in the WHO Adverse Reaction Terminology (WHO-ART) guidelines, which have been used for more than 30 years for the rational coding of adverse reaction terms [16]. The conditions were evaluated by a panel of three experts: a pharmacist, a physician, and a clinical pharmacologist. In case of any disagreement between their scores, we reached a consensus of scores by discussion. They reviewed all the available information in the reports; when necessary, the reporter of ADRs was contacted for follow-up information. Only 'possible’, 'probable’, and 'definite’ ADRs that scored more than 1 were taken into consideration; the causality of these ADRs was also classified according to the methodology of Naranjo et al. [14]. For cases with sequelae and deaths, only costs incurred during hospitalization were estimated, because the treatment cannot be tracked after the patients are discharged from hospital and hidden costs are difficult to calculate.

### Grouping of cases

All the ADR cases were divided into two groups according to serious ADRs and general ADRs in *Provisions for Adverse Drug Reaction Monitoring and Reporting*[13]. Cases with general ADRs that could be rehabilitated without any treatment, other than stopping the administration of drugs or Cases that did not require hospitalization, or prolong hospitalization, but could be rehabilitated with other drugs or therapeutic, belonged to group A. Cases with serious adverse reactions according to the *Provisions for Adverse Drug Reaction Monitoring and Reporting* belonged to group B. An ADR condition belonged to group B if it (1) caused death; (2) posed a threat to life; (3) led to significant or permanent human disability or organ damage; (4) led to brief or prolonged hospitalization; or (5) led to other important medical events. Mild reactions could auto-heal after discontinuation; moderate reactions do not result in prolonged hospitalization but could be cured by other drugs/treatment; severe reactions prolong hospitalization or other important medical events.

### Measurement of direct costs

Direct costs refer to direct medical costs, which include treatment fees, inspection fees, laboratory fees, materials fees, bed charges, drug charges, nursing care, meals, and other expenses from the beginning of the ADR to the termination of treatment. Calculation of direct costs may be based on the electronic medical data accumulated in the hospital information system (HIS). The costs of group A were calculated as the sum of the sunk-cost losses and the direct costs. If an expense incurred cannot be recovered, a rational person would ignore it. This type of expenditure is called a sunk cost. From a rational perspective, a sunk cost that cannot be recovered should not affect the consumer’s purchase decision or attitude. However, previous studies have shown that in the consumer decision-making process, the sunk cost effect cannot be ignored [17,18]. Sunk costs in the research mainly refer to the remaining drug and other costs (tutelage costs, etc.) due to the withdrawal of treatment and, therefore, the inability to reuse the ADR drug prescribed for the treatment of the original illness [19,20]. Sunk cost calculation was based on wasted drug and treatment costs accumulated in the HIS. The direct costs included treatment fees, inspection fees, laboratory fees, materials fees, bed charges, drug charges, nursing care, meals, and other expenses. Transportation costs, nutrition costs of the patients, and non-project department costs (services provided by indirect departments for example the administrative and logistical departments) were not included. The actual loss to the patient was estimated considering health-care insurance coverage available. As electronic information in the HIS record had been lost or was incomplete, cost statistics were based on average direct or sunk costs calculated from available patient data. However, this research encountered very few cases of this sort.

### Measurement of indirect costs

Indirect costs of ADR treatment were calculated according to the human capital approach (based on the 2011 GDP per capita of 19907.91 yuan for Bengbu district). Only loss of working time was estimated considering the practicability of the study. The calculation formula was as follows: lost income due to hospitalization = number of days in hospital × per capita GDP/365 for Bengbu district; lost income from accompanying hospitalized patient = number of days in attendance (e.g., number of days a child was hospitalized) × per capita GDP/365 of the concerned city (only one person was reckoned for pediatric hospitalization cases); lost income for an outpatient = number of outpatient visits due to ADR × per capita GDP/730 of the concerned city (one outpatient visit regarded as 0.5 days lost). The indirect costs of groups A and B were calculated according to the formula for the cost of inpatient care. Only when some ADR cases in group A self-heal after stopping drug treatment can the indirect costs of group A (about one outpatient per case) be calculated from lost outpatient income based on actual outpatient visits. The overall socioeconomic loss includes direct, indirect, and hidden costs. Direct and indirect costs were estimated as described in this paper. However, hidden costs, representing the pain, sadness, and depression of patients, were not considered in the present study because they were too difficult to measure.

### Data sources

All ADR cases were identified from the Chinese *Adverse Drug Reaction Monitoring and Reporting* database (http://www.adr.gov.cn). To calculate the incidence of adverse reactions, we investigated the total number of hospitalized patients during the study period through the HIS.

### Statistics

Numerical data were presented as number of observations and constituent ratios; chi-square and Fisher’s exact probability were used in univariate hypothesis testing. Descriptive analysis was conducted for quantitative variables. Mean values and *X*^*2*^ test statistics were used to compare the distributions of categorical variables. Data are presented as mean (SD), and categorical variables are expressed as percentages of the total number of ADRs. *P <* 0.05 was considered statistically significant. Excel was used to create the ADR database, and the Stata11.0 software was adopted to analyze the data.

### Ethics

This study was approved by the Center of Adverse Drug Reaction monitoring, Bengbu Food & Drug Administration in China. The Research was in compliance with the Helsinki Declaration and was approved by the Ethics Committee of The First Affiliated Hospital of Bengbu Medical College.

## Results

### Pharmacoeconomic consequences of measurement of ADRs

During the study period, 2973 cases of adverse drug reactions/inpatient events were monitored and reported by the hospital’s pharmacy department. Of these, cases that scored below 1 and those whose electronic information in the HIS record had been lost or was incomplete (234 cases) were excluded. Thus, 2739 cases of ADR were included in the study. No deaths occurred during the study period. In the same period, inpatients in the hospital numbered 337,175, and all of them received drug treatment, so that the ADR rate was 0.85%. Patient ages ranged from 1 day to 99 years with an average of 48.51 ± 19.84 years (SD); 47.72% of all patients were male. The demographic characteristics of the patients in groups A, and B are shown in Table 1.

**Table 1:** The demographic characteristics of the patients in the three groups A and B

**Group**	**Age group (year)**						
	**0-15**	**16-30**	**31-45**	**46-60**	**61-75**	**>75**	**Total**
Group A	174	301	667	808	514	234	2698
Male	118	152	237	332	291	153	1283
Female	56	149	430	476	223	81	1415
Group B	2	3	5	8	6	17	41
Male	1	2	1	5	1	14	24
Female	1	1	4	3	5	3	17
Total (%)	176	304	672	816	520	251	2739
	(6.43)	(11.10)	(24.53)	(29.79)	(18.99)	(9.16)	(100.00)
Male (%)	119	154	238	337	292	167	1307
	(4.35)	(5.62)	(8.69)	(12.30)	(10.66)	(6.10)	(47.72)
Female (%)	57	150	434	479	228	84	1432
	(2.08)	((5.48)	(15.84)	(17.49)	(8.33)	(3.06)	(52.28)

The total socioeconomic loss from the 2739 cases of ADR was estimated at ¥817401.69, consisting of direct costs of ¥603252.81 and indirect costs of ¥214148.88 for the two groups. Medical insurance payments toward direct costs amounted to ¥324483.15, and the personal burden was ¥278778.49 (Table 2). As shown in Table 2, the total costs for groups A and B were estimated to be ¥529077.80 and ¥288,323.89, respectively. The direct cost of group A was ¥335820.06 (63.47% of the total cost) and the drug cost was ¥178656.19 (33.77%). The direct cost of group B was ¥267432.75 (92.75% of the total cost) and the drug cost was ¥183886.91 (63.78%; see Table 2). The average cost per patient was ¥298.43. with the average direct costs per patient ¥124.47 and ¥6522.75 and the average total costs per patient ¥196.10 and ¥7032.29 in groups A and B, respectively. Finally, the ratios of ADR insurance reimbursements to total losses were 65.23% and 39.42% for groups A and B, respectively.

**Table 2:** Sum of the cost estimates for ADR cases over 5 years of the two groups

**Group**	**Direct cost over 5 years (¥)**		**Sum of indirect costs over 5 years**	**Sum of Medicare payments (¥) and proportion (%) over 5 years**	**Total direct costs and average direct costs per patient**	**Total costs and average costs per patient**	**Total costs, direct costs, and indirect costs from the 2739 cases**
	**Sum of drug charges**	**Sum of costs of treatment and inspection**					
			**(¥)**		**(¥)**	**(¥)**	**(¥)**
Group A	178656.19	157172.62	193235.84	219061.13 (65.23 %)	335820.06, 124.47	529077.80, 196.10	817401.69, 603252.81, 214148.88
Group B	183886.91	83545.92	20890.99	105422.02 (39.42 %)	267432.75	288323.89, 7032.29	
					6522.75		

### Evaluation of adverse drug reactions

The relationship between adverse drug reaction rates and patient gender is shown in Table 3. The table shows that the adverse reaction incidence in female patients is significantly higher than in male patients (*P <* 0.05).

**Table 3:** Relationship between gender and adverse reaction of patient

**Sex**	**ADR related**	**Not ADR related**	**Total**
Male			
No.	1307	167109	168416
Rate (per 1000 patients)	7.76	992.24	
Female			
No.	1432	167327	168759
Rate (per 1000 patients)	8.49	991.51	
Total	2739	334436	337175

There were significant differences in ADR incidence between different age groups of patients. ADR incidence per thousand patients increased from 4.48 (0–15 age group) to 23.73 (61–75 age group), and was statistically analyzed. The display group (61–75 years) showed significantly higher incidence than other age groups (*χ*^2^ = 12000, *df* = 5, *P* < 0.0001). Table 4 shows that the ADR incidence in old-age patients was significantly higher than in other age groups (*P* < 0.0001).

**Table 4:** Correlation of adverse drug reaction (ADR)-related hospitalizations in China by age group

**Variable**	**Age group (years)**						
	**0-15**	**16-30**	**31-45**	**46-60**	**61-75**	**>75**	**Total**
No. of patients (ADR-related hospitalization	176	304	672	816	520	251	2739
Rate (per 1000 patients)	4.48	4.22	6.18	11.81	23.73	22.35	8.50
No. of patients (ADR induced by antibiotics)	92	125	262	248	152	77	956
Rate (per 1000 patients)	2.34	1.74	2.41	3.59	6.94	6.86	2.97
No. of patients (non-ADR-related hospitalization)	39067	71690	108031	68303	21396	10979	319466
Rate (per 1000 patients)	995.52	995.78	993.82	988.19	976.27	977.65	991.50
Total no. of patients	39243	71994	108703	69119	21916	11230	322205

Antibiotics were the most frequent cause of ADRs, with 957 patients (34.94%) experiencing an ADR associated with this drug class, followed by digestive system drugs with 463 patients (16.90%) and traditional Chinese medicines (TCM) with 427 patients (15.59%). The top ten drugs that caused adverse reactions are listed in Table 5 according to WHO Adverse Reaction Terminology (WHO-ATC) drug coding [15,16]. Levofloxacin was the most frequently reported individual drug (192 patients, 7.01%). Table 4 shows 956 cases of antibiotic-related ADRs, accounting for 34.90% of the total number of ADRs. According to the statistical analysis results, the display group (61–75 years) showed significantly higher incidence than other age groups (*P* < 0.0001).

**Table 5:** Top twenty pharmaceuticals that caused ADRs

**Generic names**	**ATC code**	**No.**	**Percent (%)**	**Ranking**
Levofloxacin	J01MA01	192	7.01%	1
Aminomethylbenzoic acid	B02AA01	176	6.43%	2
Vitamin K1	B02BA01	89	3.25%	3
Cefathiamidine	J01DB01	52	1.90%	4
Mezlocillin	J01CE01	48	1.75%	5
Cefoperazone	J01DD01	47	1.72%	6
Ciprofloxacin	J01MA01	45	1.64%	7
Shenmai injection	TCM	42	1.53%	8
Compound amino acid	B05B002	40	1.46%	9
Iopromide	V08AB02	40	1.46%	9
Sodium Aescinate for injection	TCM	36	1.31%	10
Diammonium glycyrrhizinate	A05BA08	33	1.20%	11
Cefoperazone sodium and sulbactam sodium for injection	J01DD12	33	1.20%	11
Pefloxacin	J01MA03	33	1.20%	11
Shengmai injection	TCM	31	1.13%	12
Shuganning injection	TCM	31	1.13%	12
Tanreqing injection	TCM	31	1.13%	12
Cefoxitin	J01DC01	31	1.13%	12
Ornithine aspartate	A05BA	30	1.10%	13
Shuanghuanglian for injection	TCM	28	1.02%	14
Tiopronin	A05BA	27	0.99%	15
Matrine	A05BA	27	0.99%	15
Cefotiam	J01DC07	27	0.99%	15
Ceftazidime	J01DD02	27	0.99%	15
Ceftizoxime	J01DD07	25	0.91%	16
Cefotiam	J01DC07	25	0.91%	16
Enoxacin gluconate	J01MA04	25	0.91%	16
Piperacillin sodium and sulbactam sodium/three triazole	J01RA01	24	0.88%	17
Fosfomycin	J01XX01	24	0.88%	17
Azithromycin	J01FA10	23	0.84%	18
Potassium aspartate and magnesium	A12BA	23	0.84%	18
Vitamin B6	A11DB	23	0.84%	18
Aztreonam for injection	J01DF01	22	0.80%	19
Yadanzi youru zhusheye	TCM	20	0.73%	20

ADR names are coded with the preferred WHO-ART terms, which have been developed over more than 30 years to serve as a basis for rational coding of adverse reaction terms [14]. The following are some important ADRs with their incidence: skin and appendages disorders, 868 cases (31.69%); body as a whole—general disorders, 837 cases (30.56%); gastro-intestinal system disorders, 485 cases (17.71%); respiratory system disorders, 145 cases (5.29%); central and peripheral nervous system disorders, 127 patients (4.64%); heart rate and rhythm disorders, 103 cases (3.76%); vascular (extracardiac) disorders, 102 cases (3.72%); application site disorders, 23 cases (0.84%); hearing and vestibular disorders, 14 cases (5.11%); general cardiovascular disorders, 11 cases (0.40%); musculo-skeletal system disorders, 11 cases (0.40%); urinary system disorders, 5 cases (1.83%); liver and biliary system disorders, 5 cases (1.83%); and white cell and red disorders, 3 cases (1.10%).

Mild and moderate reactions accounted for 59.47% and 39.03%, respectively, of the reported cases, and only 1.5% of the reactions were deemed to be severe, as shown in Table 1. Two cases of anaphylactic shock caused extensions of hospitalization by 32 days and 30 days; the shortest case of hospital stay was one day extended. No deaths occurred, and all patients were cured. Patients cannot be tracked after discharge from hospital because the HIS records only information during hospitalization.

## Discussion

The incidence of adverse drug reactions among hospitalized patients during the 5-year period of this study was 0.81%, which was relatively low compared with other studies [21-23]. First, this retrospective study was based on a spontaneous reporting and monitoring system, where underreporting by healthcare workers is a possibility. Although spontaneous reporting is the most widely used method for routine monitoring of ADRs [24], it cannot guarantee that a particular adverse event is a true ADR. Second, it is possible that hospitalized patients affected by ADR were not included in the study. Third, the ADR definition in China could be somewhat different from the definition in other samples studied [25,26]. The Chinese definition did not include harmful reactions from normal doses of eligible drugs administered for medication. Thus, this definition excludes adverse events due to drug quality, administration with no indications, and off-label use.

This study showed that the incidence of adverse reactions of female patients was significantly higher than that of male patients, which was inconsistent with the adverse reactions study carried out by Agnes Chan in Taiwan [27]. Zhang et al. [28] retrospectively analyze 1001 cases of ADRs/events induced by traditional Chinese medicine injections and find that the incidence rate in female patients was higher than that in male patients. In the retrospective study conducted by Jiang and Kuang [29], the incidence of severe ADRs was also higher in women than it was in men. This result may be because TCM injections, used in many hospitals in China, are more commonly administered to women than men [30]. The other reason may be related to the different sensitivity levels and metabolic processes in men and women. Our results are consistent with Lee’s research finding that adverse reactions are significantly higher in patients more than 61 years than in other age groups [31]. Similar to other researchers in China [32,33], our study showed that the most common drugs involved were antimicrobial drugs. However, Jimmy Jose et al. show that the most common ADRs in 2006 were antineoplastic agents, antiepileptics and antibiotics ranked only third [25]. In China, more than 60% of inpatients in hospitals receive antibiotics. This may be due to the widespread clinical use of antimicrobial drugs.

However, developing countries have witnessed a growing number of ADR studies, following the damaging effects of the disease on the socioeconomic progress of those countries. A prospective analysis of 18,820 hospitalized patients was carried out in the UK [34]. The result showed that 1225 hospital admissions related to an ADR, showing a prevalence rate of 6.5%, with ADR directly leading to admission in 80% of the cases. The median bed stay was 8 days, accounting for 4% of the hospital bed capacity. The projected annual cost of such admissions to the NHS is ￡466 million (€706 million, $847 million). The results of a prospective study in France showed that hospitalization costs per ADR patient were about € 11,357.00 [35]. This study found that the total socioeconomic loss from the 2739 cases of ADR was ¥817401.69 and that the cost per ADR patient was approximately ¥298.43. This cost is far below the level of developed countries, which may reflect the lower medical care and drugs charges in China. For these calculations, the RMB exchange rate against the US dollar is approximately 6.061, while the euro exchange rate is approximately 8.197 [36]. The indirect costs were far less than the direct costs because chaperone charges and intangible losses were not included in the data. Therefore, the true value might be far greater than the calculated value in the present study.

The longest period of hospitalization for the most serious ADR case was 32 days, the shortest period was 2 days, and the average was 10.8 days. The highest payment was ¥39,873.78, and the lowest was ¥251.13. Although severe cases were comparatively few, direct costs accounted for 53.14% of the total loss from direct costs. This should be a focus area for future research. The costs of the two groups were significantly different, indicating that drug administration and treatment should be withdrawn promptly after an ADR occurrence, because most ADRs can be cured by withdrawal of the suspected drug [37]. To reduce and control serious ADR incidence, we require detailed knowledge of the patient’s history regarding food and drug allergy, smoking and drinking, and disease and medication. In addition, we also need to strengthen drug monitoring, especially for patients with allergic conditions. This would greatly reduce the associated costs, and the economic burden on the individual patient, the health-care sector, and medical institutions. At the same time, we can avoid or control ADR costs by identifying which of the ADR-related costs are relatively greater. This would be the topic of a future research. There is also a great cost difference between the two groups, suggesting that the occurrence of ADRs should promptly occur after withdrawal and treatment. Moreover, the reduction and control of the high ADR rate can greatly reduce costs, both for the patient and for the health department, thus mitigating the economic burden of medical institutions. Therefore, how to optimize ADR social costs is the most important issue.

As shown in Table 2, the proportion of Medicare payments (in China, people who pay medical insurance on time are covered by Medicare) increased with the reduction of medicine costs as a proportion of direct costs. This shows that drugs used for the treatment of ADRs were largely outside the scope of Medicare, which caused more ADRs. The higher the proportion of drug costs, the heavier the personal burden. Because inspection costs increased in groups B, and the proportion of drug costs decreased, the proportion of Medicare payments increased. However, since drug choice policies and number of patients covered by Medicare differ across hospitals, the actual proportions are not consistent with those in this study. According to the current ADR definitions and relief systems in our country, personal injury and economic loss caused by ADR would have to be borne by patients in most cases, though they could get a certain amount of health insurance compensation. However, because health insurance coverage is limited at present, the number of insured and amount of compensation are inadequate.

With price reforms in the current health system under way, the overall direct economic ADR losses of our hospitals will decrease by 10.54%. Therefore, drug price reduction would not only alleviate the problem of the high medical treatment costs but also help reduce the direct economic losses caused by ADRs.

The ADR evaluation of this study were similar to those reported by most domestic studies. However, the study focused on a single district. Cost estimates of multi-center studies would greatly enhance credibility. We hope the ADR monitoring departments of hospitals would initiate efforts to provide an economic basis for the establishment of effective ADR relief measures in China.

## Conclusions

The costs of especially severe cases of ADR cannot be ignored. In the First Affiliated Hospital of Bengbu Medical College, 0.13% of patients were identified as suffering from ADRs associated with higher direct costs largely due to extended hospitalization. Although severe ADR incidence was lower, the cost of treatment for severe ADRs was relatively high. Therefore, we should focus on the prevention of severe ADRs through ADR monitoring programmes. Further, for ADRs that automatically improve after drug withdrawal, the cost is relatively low, suggesting that stopping the medicine altogether is the best treatment for these kinds of ADRs.

## Competing interests

All authors have completed the Unified Competing Interest form at http://www.icmje.org/coi_disclosure.pdf (available on request from the corresponding author) and declare: no support from any organisation for the submitted work; no financial relationships with any organisations that might have an interest in the submitted work in the previous 3 years; no financial relationships with any organizations that might have an interest in the submitted work in the previous 3 years; no other relationships or activities that could appear to have influenced the submitted work.

## Authors’ contributions

QPS and XDH: study design and conception, data/statistical analyses, drafting the manuscript; FD and YL: study design, data/statistical analyses; MLY, JXZ and SQZ: data collection and statistical analyses. All authors contributed to writing the final manuscript. All authors read and approved the final manuscript.

## Pre-publication history

The pre-publication history for this paper can be accessed here:

http://www.biomedcentral.com/1472-6963/14/73/prepub
